# Ganglionar tuberculosis infection evolving to hemophagocytic lymphohistiocytosis after anti-programmed cell death 1 treatment for high-risk melanoma: a case report

**DOI:** 10.1186/s13256-021-02900-8

**Published:** 2021-07-08

**Authors:** Cesar M. Costa, Luiza L. Gadotti, Maria C. Seiwald, Alessandra C. R. Salgues, Fernando Ganem, Ellen C. T. Nascimento, David E. Uip, Celso Arrais-Rodrigues, Rodrigo R. Munhoz

**Affiliations:** 1 Oncology Center, Hospital Sírio Libanês (HSL), Rua Dona Adma Jafet, 91, 2nd floor, Building A, São Paulo, SP 01308-050 Brazil; 2 Division of Hematology, Universidade Federal de São Paulo (UNIFESP), São Paulo, SP Brazil

**Keywords:** Tuberculosis, Ganglionar tuberculosis, Hemophagocytic lymphohistiocytosis, Melanoma, Immune-related adverse events, Immune checkpoint inhibitors, Anti-PD-1, PD-1 blockade, Nivolumab, Immunotherapy

## Abstract

**Background:**

Hemophagocytic lymphohistiocytosis is a rare, potentially fatal syndrome of immune hyperactivation. Here we describe a ganglionar tuberculosis evolving to hemophagocytic lymphohistiocytosis following adjuvant immunotherapy in a melanoma patient.

**Case presentation:**

A 76-year-old Caucasian male with melanoma started with fever, diffuse petechiae, splenomegaly, anemia, thrombocytopenia, hypofibrinogenemia, and hyperferritinemia 2 months following completion of adjuvant treatment with nivolumab. Positron emission tomography scan showed significant hypermetabolism in cervical, supraclavicular, mediastinal, and abdominal lymph nodes. Bone marrow aspiration demonstrated no alterations, except for a hypercellular pattern. Dexamethasone and intravenous immunoglobulin were started owing to suspicion of hemophagocytic lymphohistiocytosis. Core biopsy of the infracarinal lymph node revealed a chronic granulomatous inflammation and caseous necrosis, with positivity for *Mycobacterium tuberculosis* by polymerase chain reaction, and treatment for ganglionar tuberculosis was started.

**Conclusion:**

This case highlights the challenges involving programmed cell death 1 blockade in high-risk melanoma, in which infections, lymphoproliferative disorders, and sarcoidosis can mimic disease progression and trigger immune-related adverse events.

## Background

Melanoma is a common malignancy, being the fifth most prevalent in men and the sixth in women in the USA [1]. While curable through surgical procedures when diagnosed at early stages, the prognosis for those with locoregional or distant disease has been historically ominous. Immune checkpoint inhibitors (ICIs) have resulted in favorable outcomes in patients with melanoma both in the metastatic and adjuvant settings, and the use of neutralizing antibodies targeting programmed cell death 1 (PD-1) and cytotoxic T-lymphocyte-associated protein 4 (CTLA-4) are now clinically approved in many countries [2]. As a result of the widespread use of ICIs, immune-related adverse events (irAE) have become frequent complications, often with challenging presentations and clinical courses. Hemophagocytic lymphohistiocytosis (HLH) is a rare, potentially fatal inflammatory syndrome, characterized by hyperactivation of histiocytes and lymphocytes. Conditions associated with secondary HLH are infections, malignancies or rheumatologic disorders [3]; HLH induced by ICI, however, has been rarely reported. Here we describe a ganglionar tuberculosis (TB) infection reactivation evolving to HLH in a patient treated with adjuvant nivolumab for high-risk cutaneous melanoma.

## Case presentation

A 76-year-old Caucasian male patient with no comorbidities living in Brazil was diagnosed in 2017 with a superficial spreading melanoma on his trunk, Clark level IV, Breslow 5.0 mm, with 3.6 mitoses/mm^2^, ulcerated, resected with involved margins. Initial 18-fluorodeoxyglucose positron-emission tomography (18F-FDG PET-CT) scan showed no signs of distant disease. A wide lesion excision of the primary was performed, and sentinel lymph node biopsy confirmed the presence of nodal micrometastasis (01/01), hence with a pT4b pN1a M0 initial stage (stage IIIC-AJCC 8th edition). The decision was made to proceed with adjuvant treatment with nivolumab 3 mg/kg administered every 14 days for 12 months. No clinically relevant adverse events were reported during this period.

**Fig. 1:**
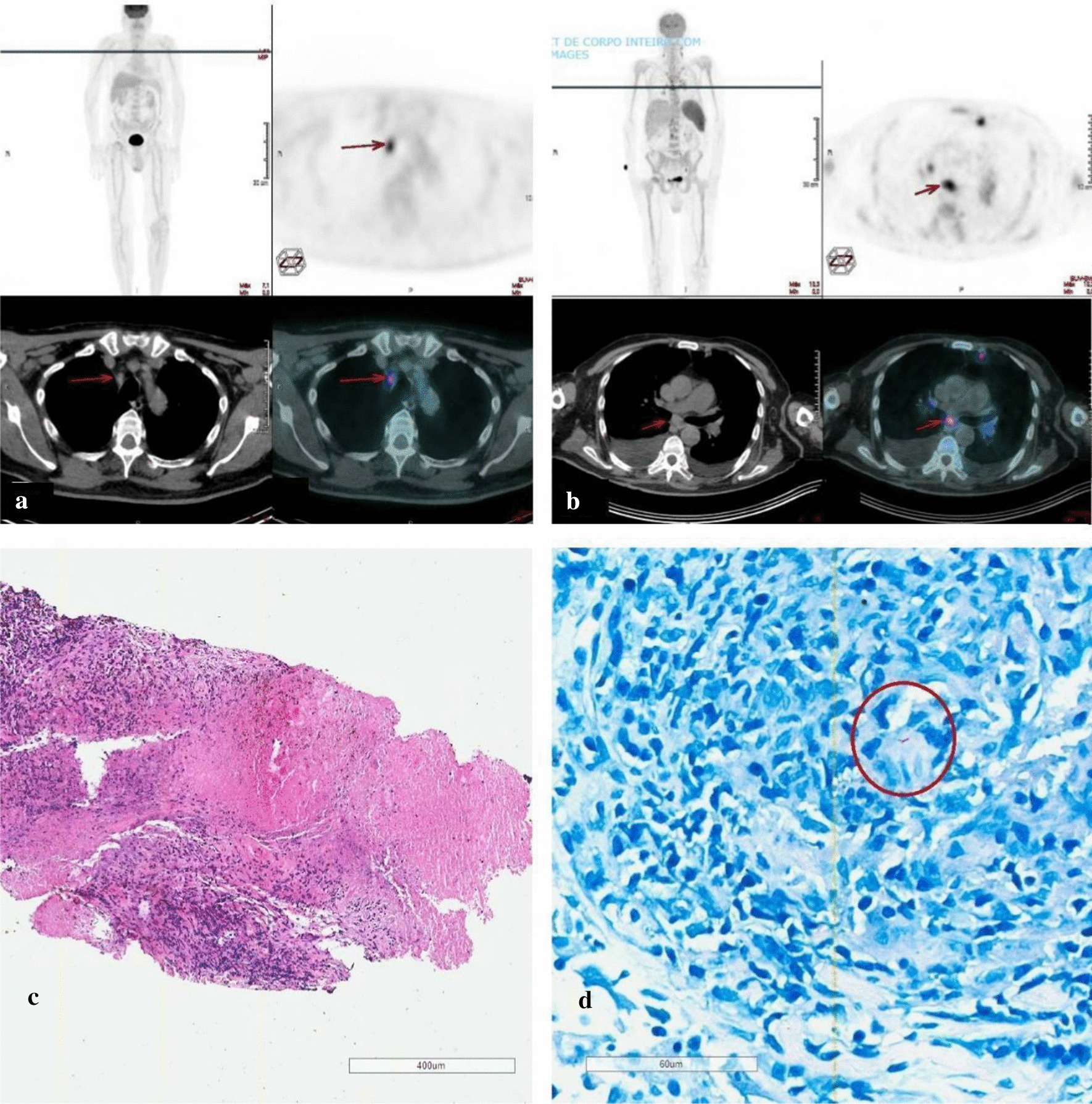
**a** Initial staging PET-CT indicating mild FDG uptake in right upper paratracheal lymph node (red arrow). Fine-needle aspiration (FNA) showed lymphoid hyperplasia, with no evidence of malignancy. **b** Two months after nivolumab completion, PET-CT scan demonstrating a hypermetabolic infracarinal lymph node (red arrow) with generalized lymphadenopathy (cervical, mediastinal, and abdominal), and splenomegaly. **c** Hematoxylin and eosin (H&E) staining showing epithelioid cell granuloma with central coagulation necrosis. **d** Ziehl–Neelsen staining showing acid-fast bacilli (red circle)

Two months following nivolumab completion, the patient presented with weakness, inappetence, daily fever (38.0 ℃), night sweats, weight loss, diffuse petechiae, and splenomegaly. He denied any recent travels, new drug usage, or contact with symptomatic individuals. Initial investigation revealed anemia (8.5 g/dl), thrombocytopenia (68.000/m^3^), hypofibrinogenemia (138 mg/dl), and hyperferritinemia (20.406 µg/l). PET-CT detected intense 18F-FDG uptake in the spleen and in the cervical, supraclavicular, mediastinal, and abdominal lymph nodes (Fig. 1). Bone marrow aspiration indicated a hypercellular marrow with no other alterations. The patient started dexamethasone and intravenous immunoglobulin because of HLH diagnosis (five of eight criteria for this syndrome fulfilled).

No infection was identified after upfront extensive laboratory workup and cultures. Due to the increased 18F-FDG uptake (SUVmax 14.8) in an infracarinal lymph node, a core biopsy was performed, having metastatic melanoma, hematologic malignancy, or even rheumatologic granulomatous disorders (sarcoidosis) as differential diagnoses. However, epithelioid cell granuloma with central coagulation necrosis was observed in hematoxylin and eosin (H&E) staining (Fig. 1), and *Mycobacterium tuberculosis* (Mtb) was identified by polymerase chain reaction (PCR). Treatment for ganglionar TB with daily oral combination of rifampicin (R) 600 mg, isoniazid (H) 300 mg, pyrazinamide (Z) 1600 mg, and ethambutol (E) 1100 mg was started. The patient received the RHZE regimen for 2 months, followed by 4 months of RH doublet, completing a 6 months of antituberculous therapy. The patient’s clinical status improved significantly, with findings suggestive of HLH remission. The patient has been on clinical follow-up for 40 months, and no melanoma recurrence was detected.

## Discussion

This case presents unusual features, with a tuberculosis reactivation following adjuvant treatment for stage III melanoma associated with HLH, unclear if an irAE or secondary to the granulomatous process. There are reports suggesting that nivolumab could potentially activate latent TB [4, 5]. TB complicated with HLH has also been described [6, 7]. Furthermore, severe HLH as an irAE in a melanoma patient treated with dual checkpoint blockade was recently documented [8]. However, the relationship of these three rare manifestations together has not yet been established.

TB is most often characterized by pulmonary involvement, while extrapulmonary disease accounts for only 20% of all TB manifestations, and its association with immunosuppression such as human immunodeficiency virus (HIV), hepatitis, diabetes, alcohol abuse, drug addiction, and transplant recipients is well documented [9]. However, these conditions were all absent in this case. The only risk factor associated with Mtb infection is Brazil’s endemic area, where it accounts for the highest number of TB cases in the Americas according to the World Health Organization (WHO) [10]. TB should always be considered among the differential diagnoses in endemic areas, even when rare extrapulmonary manifestations occur.

The diagnosis of HLH is particularly challenging because symptoms are nonspecific, usually associated with a devastating hematological disorder from an uncontrolled immune activation, whose features overlap with other causes of severe illness, including sepsis and hematologic malignancies [11]. The classification of HLH relies on eight diagnostic criteria, of which five or more must be met: fever (> 38 °C); splenomegaly; cytopenias affecting two or more cell lines (hemoglobin <  9 g/dl, platelets < 100 × 10^3^/ml, neutrophils < 1 × 10^3^/ml), hypertriglyceridemia (fasting > 265 mg/dL), and/or hypofibrinogenemia (< 150 mg/dL), hemophagocytosis in bone marrow, spleen, lymph nodes, or liver; low or absent natural killer (NK) cell activity; ferritin > 500 ng/ml; elevated soluble CD25. Thus, considering HLH in the differential diagnosis requires a low threshold for suspicion [12].

The main mechanism of TB leading to HLH remains unclear. Levels of proinflammatory cytokines are higher in TB patients than in healthy individuals. Moreover, Mtb is supposed to act as a TH1-mediated cytotoxicity inducer, leading to HLH-related symptoms explained by the activating macrophages and NK cells in the inflammatory context [13]. Investigators of a multicenter retrospective cohort that included 312 adult patients with reactive HLH over a 6-year period observed that hematologic malignancies are the main condition associated with HLH, especially non-Hodgkin lymphomas (56%); Mtb infection was reported only in 7.4% of HLH cases [14].

Mtb infections have also been identified following exposure to anti-PD-1 monoclonal antibodies [15] used as monotherapy or in combination with anti-CTLA-4 agents [16]. A retrospective study evaluated the development of TB in 1144 patients with malignancies after ICI (pembrolizumab, nivolumab, or atezolizumab) treatment. Lung cancer (*n* = 796, 69.6%), melanoma (*n* = 115, 10.1%) and lymphoma (*n* = 85, 7.4%) were the most prevalent cancers. Pembrolizumab (*n* = 612, 53.5%), nivolumab (*n* = 474, 41.4%), and atezolizumab (*n* = 58, 5.1%) were the most frequent therapies. In this cohort, three patients with advanced lung cancer developed pulmonary TB, and the overall incidence rate of TB was 394.4 cases [95% confidence interval (CI) 100.3–1,073.4] per 100,000 person-years [17]. Despite these results, the retrospective design and the small number of patients are insufficient to draw precise conclusions.

The current use of ICI in the clinical practice is certainly one of the most important approaches that emerged in oncology over the past decade. Due to its increasing use, ICI toxicities have been better identified, most occurring within 6 months after initial exposition, and becoming less frequent following ICI discontinuation [18]. As PD-1 blockade becomes more globally prescribed in TB-endemic regions, it is possible that TB-related adverse events in cancer immunotherapy context may increase. Unlike immunosuppressive biologic therapies [tumor necrosis factor (TNF)-alpha inhibitors] used for the treatment of autoimmune disease, very few investigational protocols incorporating anti-PD-1 or anti-PD-L1 therapy require TB screening [5]. There is no strong recommendation whether cancer patients should be screened for latent or active TB and, if positive, receive preventive chemoprophylaxis. According to the WHO guidelines, TB screening is not recommended for those with malignancies because of the lack of evidence [19]. However, a systematic review and meta-analysis quantified the risk of active Mtb infection in cancer patients, including 23 studies with more than 300,000 patients. Despite the methodological bias, this study indicates that individuals with hematologic malignancies, metastatic head and neck squamous carcinoma, and lung cancer had a greater rate of developing active Mtb infection versus those without cancer, suggesting that TB screening and chemoprophylaxis would be beneficial in this population [20].

## Conclusion

The diagnosis of ganglionar TB reinforces the importance of a careful evaluation of patients using immunotherapy and the consideration of this diagnostic hypothesis in residents of endemic areas for TB. This case highlights the challenges involving clinical use of anti-PD-1 agents, especially in the context of high-risk cutaneous melanoma, in which infections, lymphoproliferative disorders, and even sarcoidosis can mimic disease progression. Furthermore, irAE is becoming more evident owing to the increased use of immunotherapy, but many clinical conditions are still complex, especially when associated with infectious diseases. To our knowledge, this is the first case report of an Mtb infection evolving to HLH after anti-PD-1 treatment for high-risk melanoma.

## Data Availability

Not applicable.

## Abbreviations

AJCC: American Joint Committee on Cancer
PD-1: Programmed cell death 1
E: Ethambutol
FDG: Fluorodeoxyglucose
FNA: Fine-needle aspiration
H: Isoniazid
H&E: Hematoxylin and eosin
HLH: Hemophagocytic lymphohistiocytosis
ICI: Immune checkpoint inhibitor
irAE: Immune-related adverse event
Mtb: *Mycobacterium tuberculosis*
PCR: Polymerase chain reaction
PET: Positron emission tomography
R: Rifampicin
SUVmax: Maximum standardized uptake value
TB: Tuberculosis
TNF: Tumor necrosis factor
WHO: World Health Organization
Z: Pyrazinamide
